# Transcriptome-based immune subtypes reveal the heterogeneity of tumor microenvironment in pediatric B-cell acute lymphocytic leukemia

**DOI:** 10.3389/fonc.2025.1643160

**Published:** 2025-09-10

**Authors:** Hongbin Meng, Xiaoxia Li, Wenyi Hou, Jinqiao Li, Yueyue Fu

**Affiliations:** Department of Hematology, The First Affiliated Hospital, Harbin Medical University, Harbin, Heilongjiang, China

**Keywords:** B-cell acute lymphoblastic leukemia, pediatric, RNA sequencing, tumor microenvironment, immune

## Abstract

**Background:**

Increasing evidence highlights the important role of the tumor microenvironment (TME) in B-cell acute lymphocytic leukemia (B-ALL). Our study aimed to stratify B-ALL based on immune signatures, thus helping to clinically predict prognosis and guide treatment.

**Methods:**

Two cohorts of pediatric B-ALLs were included in this study, one from the GEO database (n = 136) was used to establish consensus clustering algorithm to stratify B-ALLs based on immune-related genes (IRGs), and the other from our cohort (n = 73) was used to validate the universality of established clustering algorithm. To elucidate the characteristics of each subtype, the prognosis, immune features, clinical information and genetic abnormalities were explored.

**Results:**

Based on the expression of 1315 IRGs, B-ALLs were classified into five distinct immune subtypes. Cluster1 had the favorable prognosis while cluster 2–5 had relatively unfavorable prognosis. Cluster 1 was strongly associated with clinical information indicative of a favorable prognosis [e.g. low white blood count (WBC) level] relative to cluster 2-5. In term of immune features, cluster 5 were characterized by high expression of multiple immune checkpoint genes [e.g. B and T lymphocyte attenuator (*BTLA*), cytotoxic T-lymphocyte-associated protein 4 (*CTLA4*), and T cell immunoreceptor with Ig and ITIM domains (*TIGIT*)]. Cluster 3 and 4 exhibited significantly downregulation of antigen processing and presentation and cytokine-cytokine receptor interaction, respectively. In terms of genetic abnormalities, cluster 1, 2 and 3 demonstrated a high incidence of *ETV6-RUNX1* fusion, *NRAS* mutation and *KRAS* mutation, respectively.

**Conclusions:**

Our study identified five immune subtypes that associated with distinct biological aberrations and clinical behaviors, which help us better understand the heterogeneity of TME and may provide valuable information for the precision therapy of pediatric B-ALL.

## 1 Introduction

B-cell acute lymphoblastic leukemia (B-ALL) is the most common hematological malignancy among children, accounting for about 25% of all pediatric cancers (1). Advances in conventional therapy over the past decades led to a striking improvement in the survival of pediatric B-ALL patients (>90% five-year survival) (2). Regrettably, approximately 10% patients with B-ALL still suffer from refractory/relapse (R/R), indicating the high heterogeneity of B-ALL (3).

Of note, with the current high survival rate of pediatric B-ALL, it is difficult to improve the patient survival with only conventional chemotherapy, which has reached its maximum of tolerance and could no longer be pushed to improved patient outcomes (4, 5). Immunotherapy, an emerging novel treatment modality, has demonstrated to significantly improve the response rate and outcomes in patients with R/R B-ALL (6, 7). Based on promising efficacy of immunotherapy in patients with R/R B-ALL, immunotherapy has been incorporated into the frontline therapy for this disease (8, 9). The considerable success of immunotherapy has highlighted the essential role of the tumor microenvironment (TME) in B-ALL (10). Accumulating evidence demonstrates that TME contributes to the development and progression of cancer, and there are wide variations in the responses to immunotherapy and prognosis of ALL patients with various TME (11, 12). For example, high expression level of PD-1 on the surface of T cells in pediatric B-ALL patients contributes to immune escape, which is associated with an inferior prognosis (13). Excess T regulatory cells play a key detrimental role on the therapeutic effect of blinatumomab (a CD3/CD19-directed bispecific T-cell engager molecule) (14). In addition, previous studies revealed the underlying mechanism for leukemia relapse involves immune escape of leukemia cells, and the gene expression profile of the relapsed leukemia is highly enriched in immune-related processes (15, 16). Considering the importance and heterogeneity of TME in B-ALL, it makes sense to identify B-ALL subtypes associated with the different immune signatures.

**Figure 1:**
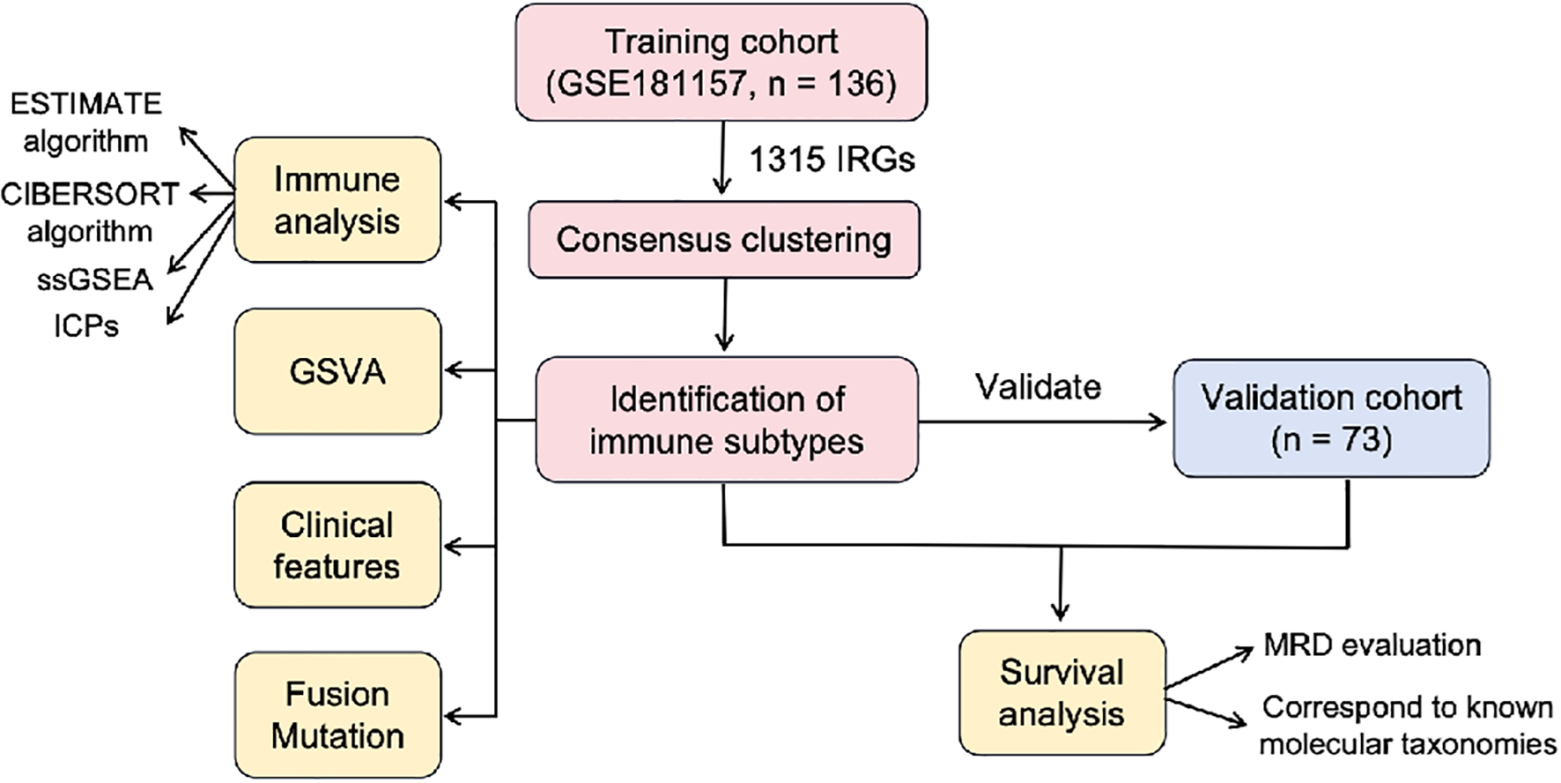
The flowchart of this study. IRGs, immune-related genes; GSVA, gene set variation analysis; ssGSEA, single-sample gene set enrichment analysis; MRD, minimal residual disease.

In our study, using the unsupervised clustering method, we attempted to classify B-ALL into distinct immune subtypes based on the expression profiles of immune-related genes (IRGs) in the training cohort, and validated the clustering approach in a validation cohort. To investigate the potential significance of each subtype, we delved deeper into the correlation between subtypes and prognosis, immune features, gene enrichment pathways, clinical features and genetic abnormalities. **Figure 1** showed the flowchart of this study.

## 2 Method

### 2.1 Patient cohorts

#### 2.1.1 Training cohort

The transcriptome data from Gene Expression Omnibus database (n=136, GSE181157, GEO database, http://www.ncbi.nlm.nih.gov/geo/) was used as the training cohort. This GSE dataset comprised the RNA sequencing (RNA-seq) data, clinical information and genetic abnormalities of 136 pediatric B-ALL patients (**Supplementary Table S1**).

#### 2.1.2 Validation cohort

A total of 73 newly-diagnosed patients with B-ALL in the First Affiliated Hospital of Harbin Medical University between May 2019 to March 2024 was used as the validation cohort. Sufficient bone marrow (BM) from patients at diagnosis were collected for RNA-seq. Clinic data were collected from the medical records (**Supplementary Table S2**). The majority of patients (66 of 73, 89.0%) were treated with Chinese Children Leukemia Group ALL 2018 protocol (CCLG-ALL-2018). The remaining 7 patients (9.6%) were untreated. No patients initially unresponsive/refractory to treatment.

### 2.2 RNA-seq and bioinformatic analyses

RNA was extracted from BM samples using the mini AllPrep DNA/RNA kits (Qiagen, Germantown, MD, USA). High quality RNA was subjected to library preparation using the Illumina TruSeq stranded mRNA kit according to the manufacturer’s recommendations with input 500 ng of total RNA. Libraries are sequenced on the HiSeq 4000 (Illumina, San Diego, CA, USA) using paired-end sequencing (2x150 bp). Quality control checks of the sequencing raw data was conducted with FastQC, while the raw reads were filtered with Trimmomatic software. Filtered reads were mapped to the human reference genome GRCh38 by STAR software version 2.1.0. Gene expression estimates were calculated using HTSeq count version 0.6.1. FPKM values obtained from normalization of the count matrix were used as the gene expression matrix for downstream analysis (17).

### 2.3 Acquisition of immune-related genes

A total of 1793 IRGs were obtained from the Immunology Database and Analysis Portal (ImmPort) database (https://immport.niaid.nih.gov) for analysis. The intersection of the IRGs and the gene expression profiles of training and validation cohorts then yielded 1315 genes that were included in our study.

### 2.4 Consensus clustering

Based on the expression patterns of 1315 IRGs, we used “ConsensusClusterPlus” package for unsupervised clustering on 136 samples from training cohort to identify different immune subtypes. Samples were classified into clusters by using the pearson distance metric and the hierarchical clustering algorithm with setting from 2 to 6. Each bootstrap contained around 80% of the samples, compiling the results for 500 bootstraps. The optimal value for the clustering number was determined by analyzing the consensus matrix heatmap, cumulative distribution function (CDF) plot and Delta area plot. We validated the immune subtypes in the validation cohort using the same settings.

### 2.5 Survival analysis

The association between immune subtypes and patient outcome was analyzed. Due to the lack of prognostic information in both the training and validation cohorts, we used the available final risk results (n = 122) obtained from the MRD results of 2 time points (at day 32 of induction 1A and at the end of induction 1B) in the training cohort and available MRD results (n = 65) evaluated at the end of induction in the validation cohort to indirectly assess the prognosis of the different immune subtypes. In addition, we showed the distribution of known molecular taxonomies carrying explicit prognostic significance in different subtypes by Sankey diagram, which could also indirectly characterize the prognosis of different immune subtypes.

### 2.6 Immune analyses

The immune features among the various subtypes were investigated. The ESTIMATE algorithm was conducted to estimate the stromal score and immune score of each sample. The CIBERSORT algorithm was applied to estimate the relative proportions of 22 tumor-infiltrating immune cells in every samples. The single-sample gene set enrichment analysis (ssGSEA) algorithm was employed to calculate the score of 19 functional gene expression signatures relevant to TME cells, cellular states, physiological and pathological processes in different samples (18). Furthermore, we analyzed the difference of immune checkpoint genes (ICPs) expression between the different subtypes. The ICPs were acquired from the previous study (19).

### 2.7 Gene set variation analysis

GSVA is a non-parametric and unsupervised method for evaluating transcriptomic gene set enrichment. In this study, gene sets were downloaded from the Molecular Signatures Database (version 7.0), and each gene set was comprehensively scored by the GSVA R package. Pathways with adjusted p values < 0.05 were deemed statistically significant. Finally, the pathways we were concerned with were presented as a heatmap.

### 2.8 Statistical analysis

Statistical analysis was performed using R package (version 4.1.2). Quantitative data was compared by Kruskal-Wallis test. Categorical variables were evaluated using the Chi-square test or Fisher’s exact test followed by Bonferroni correction. All tests were two-tailed, *P* < 0.05 was considered to be significantly different. Graphs were made in Adobe Illustrator 2021 (Adobe Inc.).

## 3 Results

### 3.1 Identification of five immune subtypes through the consensus clustering

**Figure 2:**
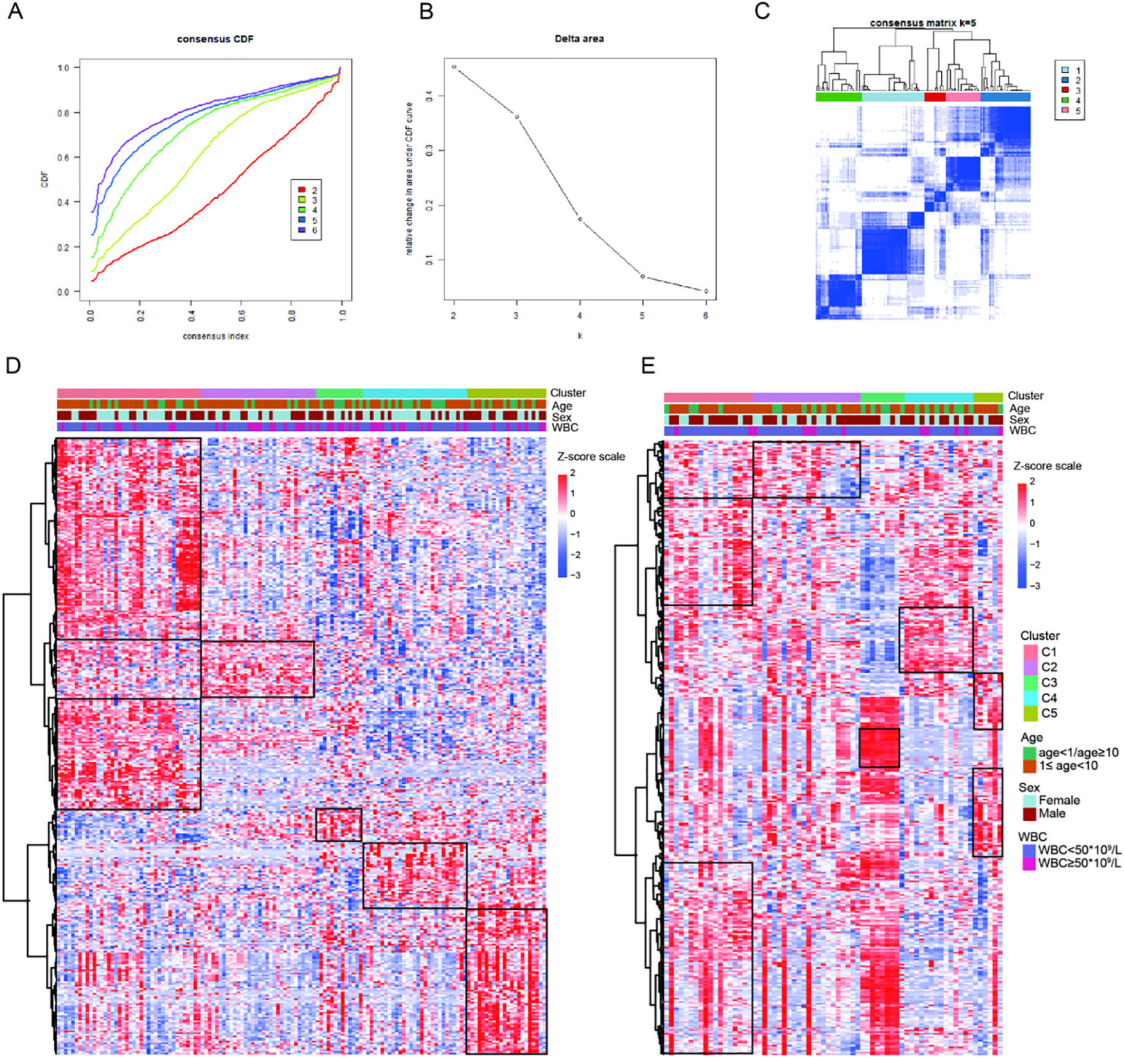
Construction of five immune subtypes through consensus clustering based on 1315 immune-related genes. **(A)** The cumulative distribution function (CDF) curves for k = 2 to k = 6. **(B)** Relative change in area under the CDF curve for k = 2 to k = 6. **(C)** The consensus matrix obtained when k = 5. Gene expression heatmap showing gene expression levels of 363 immune-related genes among the five immune subtypes in the training cohort **(D)** and validation cohort **(E)**.

Based on the expression profiles of 1315 IRGs, we conducted consensus clustering on 136 B-ALL samples from the GEO database to investigate the immune-based molecular classifications for B-ALL patients. When k = 5, the CDF and CDF Delta area plot showed greater stability, and the consensus matrix heatmap exhibited clear and distinct boundaries, thus these samples were assigned to five subtypes: cluster 1 (C1, n = 40, 29.4%), cluster 2 (C2, n = 32, 23.5%), cluster 3 (C3, n = 13, 9.6%), cluster 4 (C4, n = 29, 21.3%), and cluster 5 (C5, n = 22, 16.2%, **Figures 2A-C**). The heatmap showing the top 363 genes with the highest variance in gene expression among the subtypes demonstrated a good discrimination between the five different subtypes in the training cohort (**Figure 2D**). We applied the established unsupervised clustering algorithm to the validation cohort, where 18, 26, 9, 14, and 6 patients were classified into C1, C2, C3, C4, and C5, respectively. A similar differential expression of 363 genes was observed in the validation cohort (**Figure 2E**).

### 3.2 Prognostic differences of the five immune subtypes

**Figure 3:**
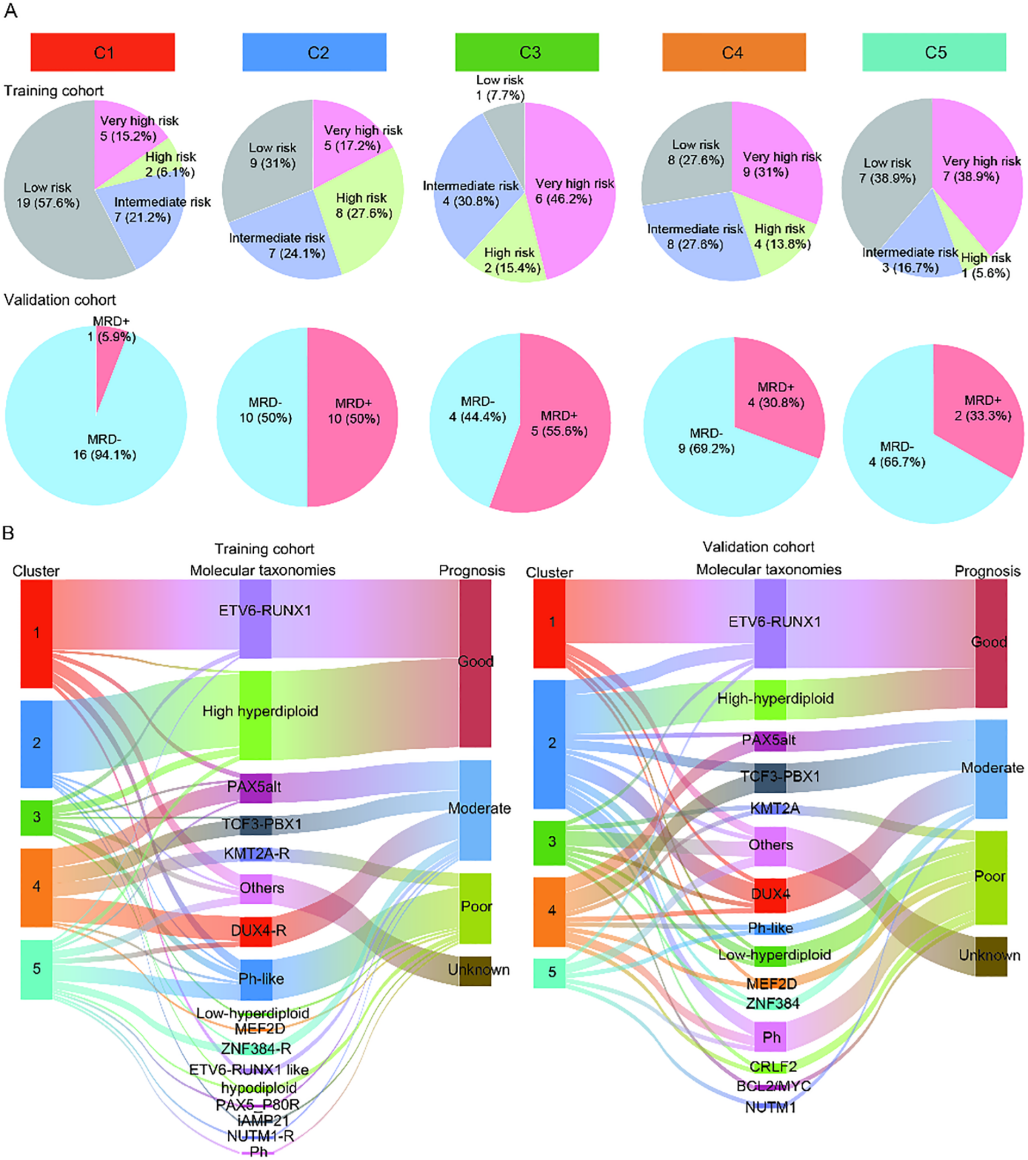
Potential prognostic value of the different immune subtypes. **(A)** The pie charts showing the percentage of patients in each subtype of MRD results in the training cohort and validation cohort. **(B)** The Sankey diagram showing the relationship between the known molecular taxonomies carrying definite prognostic significance and newly established immune subtypes in the training cohort and validation cohort.

The clinical characteristics of the patients in the training and validation cohorts were displayed in **Supplementary Table S1-2**. The median age of the patients in the training and validation cohorts was 6.1 years (range, 1.0 to 21.7 years) and 6 years (range, 1 to 16 years), respectively. In the training and validation cohorts, there were 45 (33.1%) and 13 (17.8%) patients with white blood count (WBC) ≥ 50 ×10^9^/L, respectively. Due to the lack of long-term follow-up data in the cohort, the early treatment MRD status was used as a prognostic surrogate indicator. We compared the MRD results of each subtype after induction chemotherapy. After Bonferroni correction, the significance threshold was *P*<0.01. Compared to C2-5, C1 had a markedly lower proportion of very high-risk and high-risk patients (C1 vs. C2, 21.2% vs. 44.8%, *P* = 0.047; C1 vs. C3, 21.2% vs. 61.5%, *P* = 0.009; C1 vs. C4, 21.2% vs. 44.8%, *P* = 0.047; C1 vs. C5, 21.2% vs. 44.4%, *P* = 0.082) in the training cohort, suggesting that C1 was associated with the favorable prognosis. A similar difference was observed in the validation cohort, where C1 had explicitly fewer MRD-positive patients than C2-5 (C1 vs. C2, 5.9% vs. 50.0%, *P* = 0.003; C1 vs. C3, 5.9% vs. 55.6%, *P* = 0.004; C1 vs. C4, 5.9% vs. 30.8%, *P* = 0.07; C1 vs. C5, 5.9% vs. 33.3%, *P* = 0.086). There was no significant difference in the proportion of patients at very high-risk and high-risk between C2, C3, C4, and C5 in the training cohort, indicating that there was no difference in the prognosis of patients among C2-5 (C2 vs. C3, 44.8% vs. 61.5%, *P* = 0.317; C2 vs. C4, 44.8% vs. 44.8%, *P* = 1; C2 vs. C5, 44.8% vs. 44.4%, *P* = 0.98; C3 vs. C4, 61.5% vs. 44.8%, *P* = 0.317; C3 vs. C5, 61.5% vs. 44.4%, *P* = 0.347; C4 vs. C5, 44.8% vs. 44.4%, *P* = 0.98). A similar phenomenon was observed in the validation cohort (C2 vs. C3, 50% vs. 55.6%, *P* = 0.782; C2 vs. C4, 50% vs. 30.8%, *P* = 0.275; C2 vs. C5, 50% vs. 33.3%, *P* = 0.473; C3 vs. C4, 55.6% vs. 30.8%, *P* = 0.245; C3 vs. C5, 55.6% vs. 33.3%, *P* = 0.398; C4 vs. C5, 30.8% vs. 33.3%, *P* = 0.911, **Figure 3A**). All in all, C1 had the favorable prognosis, while C2, C3, C4, and C5 had relatively dismal prognosis.

Meanwhile, we further explored the prognosis of each subtype by illustrating the distribution of known molecular taxonomies carrying explicit prognostic significance in each subtype using Sankey diagram. Of note, the majority of B-ALL patients in C1 from both the training and validation sets belonged to the *ETV6*-*RUNX1* subtype, represents a favorable prognosis (**Figure 3B**). These results reaffirmed that the prognosis of patients in the C1 was better than that of the other four clusters. In addition, from the training and validation cohorts, we also observed that many patients in C2 belonged to the high hyperdiploid subtype, suggesting that a subset of patients in C2 may have a favorable prognosis. For the C3-5, there was no correlation between clusters and known molecular taxonomies.

### 3.3 Assessment of immune features in distinct subtypes

**Figure 4:**
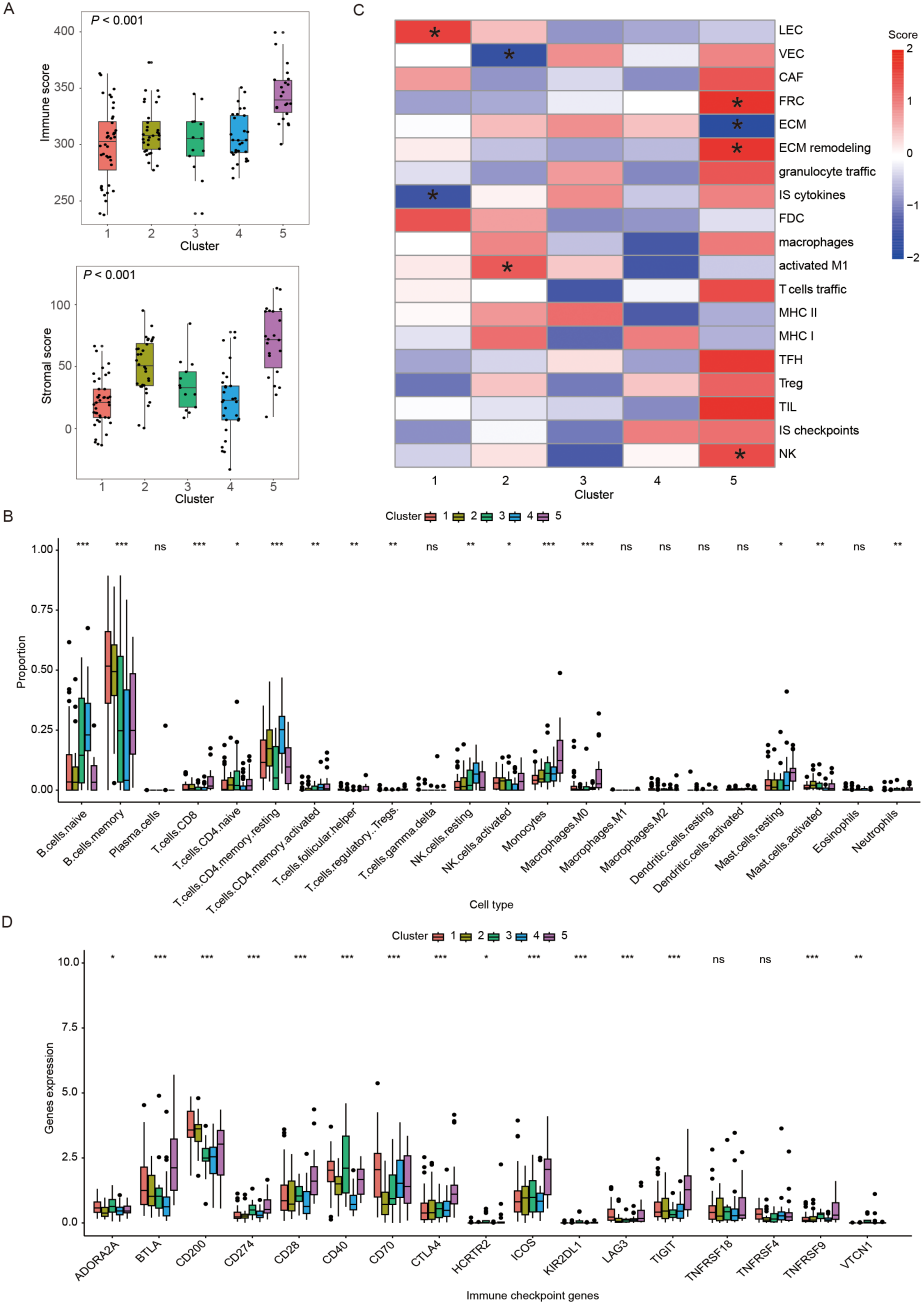
Immune landscape of different immune subtypes. **(A)** Box plots showing the immune and stromal scores of five immune subtypes. **(B)** Heatmap demonstrating scores of immune-related gene set in five immune subtypes, where * represented that the ssGSEA scores of this set in this subtype was significantly different with the other subtypes. **(C)** Box plots displaying the distribution of the 22 immune cell proportions in five immune subtypes. **(D)** Box plots exhibiting the differential expression of immune checkpoint genes among five immune subtypes. In figure **(C, D)** *, **, ***, and ns represented P < 0.05, P < 0.01, P < 0.001 and non-significant, respectively. The P-value presented in the box plots corresponded to the statistical comparison among the five immune subtypes. LEC, lymphatic endothelial cells; VEC, vascular endothelial cells; CAF, cancer-associated fibroblasts; FRC, fibroblastic reticular cells; ECM, extracellular matrix; IS: immune suppressive; FDC, follicular dendritic cells; MHC, major histocompatibility complex; TFH, follicular T-helper cells; TIL, tumor infiltrating lymphocytes; NK: natural killer cells.

To explore the possible reasons for the differential prognosis of the five immune subtypes, the TME of patients in distinct subtypes was analyzed. First, we calculated the immune score and stromal score in each sample using the ESTIMATE method, and found significant differences among these five subtypes (*P* < 0.05). The immune score, stromal score (*P* all < 0.05) were significantly higher in the C5 than those in the C1-4. In C1-4, C2 had significantly higher stromal scores than the C1, C3 and C4 (*P* < 0.05, **Figure 4A**). Then, we elucidated the immune cell infiltration by CIBERSORT analysis. Excluding low-infiltrating immune cells, we found that immune cell composition varied across the five subtypes (**Supplementary Table S3**). The TME of the C5 was enriched of more CD8 T cells, M0 macrophages, monocytes and resting mast cells as compared to other subtypes (*P* < 0.05). C4 had higher abundance of resting memory CD4 T cells but lower abundance of activated natural killer (NK) cells in comparison to other subtypes (*P* < 0.05). C2 demonstrated lower infiltration of M0 macrophages compared to other subtypes (*P* < 0.05, **Figure 4B**). Next, we used ssGSEA method to calculated the scores of some TME cells, cellular states, physiological and pathological processes. We found significant differences in some scores among five immune subtypes. Specifically, C1 had higher score in lymphatic endothelial cells (*P* < 0.05) but lower score in immune suppressive cytokines (*P* < 0.05). C2 exhibited higher score in activated M1 macrophages (*P* < 0.05) while lower score in vascular endothelial cells (VEC, *P* < 0.05). C5 showed high scores in extracellular matrix (ECM) remodeling (*P* < 0.05), fibroblastic reticular cells (FRC, *P* < 0.05) and NK cells (*P* < 0.05), and lower score in ECM (*P* < 0.05, **Figure 4C**). Finally, we compared the ICP expressions among the five subtypes. We found significant discrepancy in the expression of some ICPs among the five subtypes. Compared with other clusters, cluster of differentiation 40 (*CD40*, *P* < 0.05) was highly expressed in C4, and C5 had higher ICP expressions of B and T lymphocyte attenuator (*BTLA*), cluster of Differentiation 28 (*CD28*), cytotoxic T-lymphocyte-associated protein 4 (*CTLA*), inducible T-cell Costimulator (*ICOS*) and T-cell Immunoreceptor with Ig and ITIM domains (*TIGIT*) (*P* < 0.05, **Figure 4D**).

### 3.4 Assessment of gene enrichment pathway in distinct subtypes

**Figure 5:**
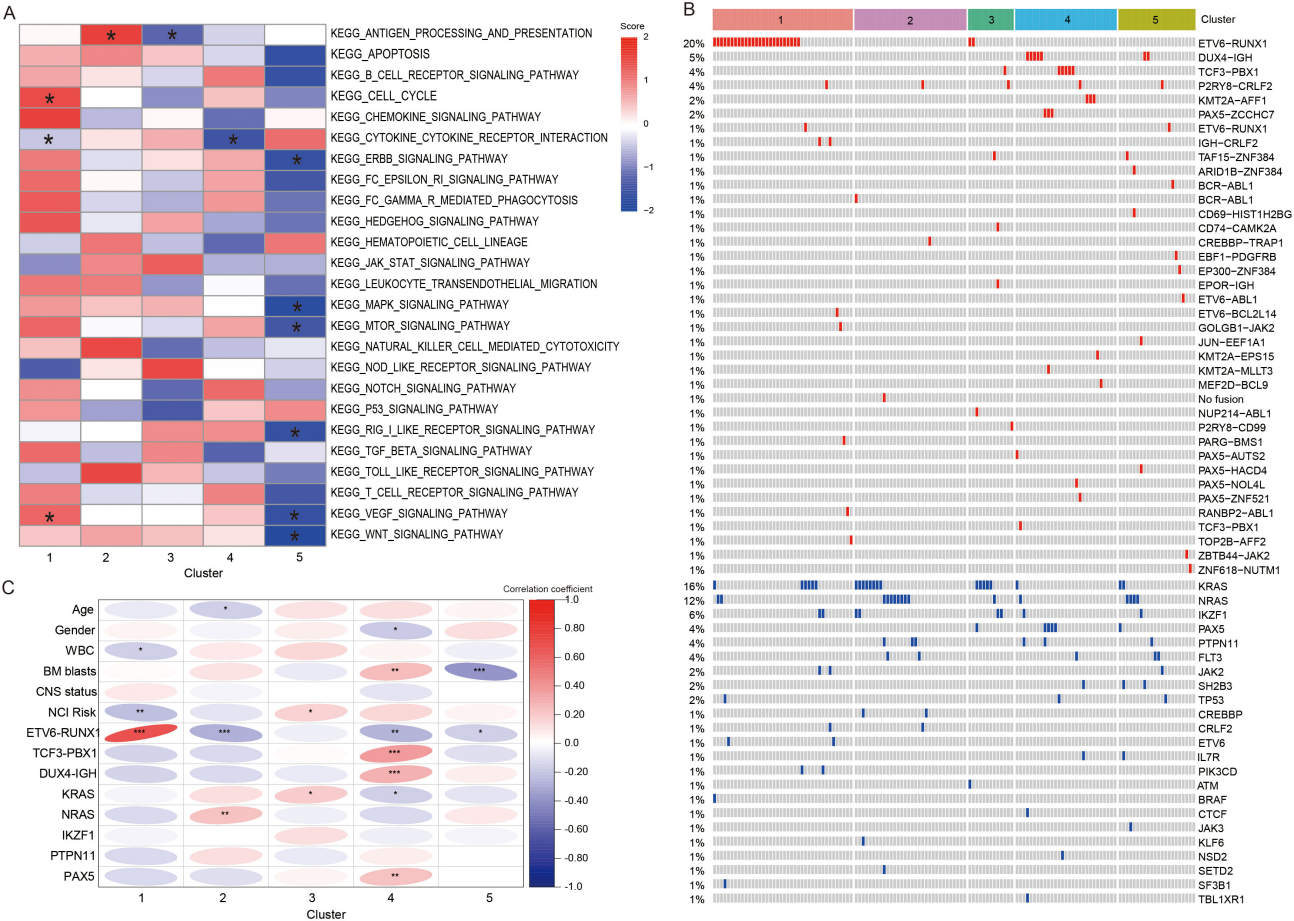
Differences in enrichment pathways, clinical characteristic, and genetic abnormalities between different clusters. **(A)** Heatmap showing the significant pathway with the GSVA scores. * represents that the GSVA scores of this pathway exhibited statistically significant differences in the specified subtype compared to all other subtypes. **(B)** Heatmap showing the fusion and mutation profiles of 5 subtypes. **(C)** The correlation heatmap demonstrating the relationship between subtypes and clinical information, fusion and mutant genes. *, **, and *** represented P < 0.05, P < 0.01, and P < 0.001. WBC, white blood cells; BM, bone marrow; CNS, central nervous system.

The GSVA algorithm revealed significant differences among five subtypes in some pathways (20). After multiple analyses, the results found that C1 showed significant activation of cell cycle and VEGF signaling pathway (*P* < 0.05). C2 displayed significant upregulation of antigen processing and presentation (APP, *P* < 0.05), whereas C3 exhibited significant downregulation of this pathway (*P* < 0.05). In C4, the suppressed genes were enriched in cytokine-cytokine receptor interaction (*P* < 0.05). C5 had 6 significantly inhibited pathways, including ERBB signaling pathway, MAPK signaling pathway, MTOR signaling pathway, RIG-I-LIKE receptor signaling pathway, VEGF signaling pathway, and WNT signaling pathway (*P* < 0.05, **Figure 5A**).

### 3.5 Assessment of genetic abnormalities in distinct subtypes

In addition, we portrayed the fusions and gene mutations of 5 subtypes (**Figure 5B**). Notably, C1 demonstrated a high incidence of *ETV6-RUNX1* fusion. Then, we screened for fusions and mutant genes carried by more than five patients in the training cohort, and explored their relationship with subtypes (**Table 1**). The results showed that five subtypes had their own significant characteristics. C1 consisted of a higher number of patients with WBC < 50 × 10^9^/L (*P* < 0.05), *ETV6-RUNX1* fusion (*P* < 0.001) or at standard risk (NCI risk, *P* < 0.01). C2 contained fewer patients aged 1–10 years (*P* < 0.05) or carrying *ETV6-RUNX1* fusion (*P* < 0.001), but contained more patients with *NRAS* mutations (*P* < 0.01). In C3, more patients were at high risk (*P* < 0.05), or carried *KRAS* mutations (*P* < 0.05). Female (*P* < 0.05) and high BM blast level (*P* < 0.01) were correlated with the C4. And, C4 had a higher proportion of patients with *TCF3-PBX1* fusion (*P* < 0.001), *DUX4-IGH* fusion (*P* < 0.001), or mutant *PAX5* (*P* < 0.01), and a lower proportion of patients with *ETV6-RUNX1* fusion (*P* < 0.05) and *KRAS* mutation (*P* < 0.05). C5 was characterized by a lower proportion of patients with high BM blast level (P < 0.001) or *ETV6-RUNX1* fusion (*P* < 0.05). Overall, the clinical indicators and genetic abnormalities in C1 were all more inclined toward a favorable prognosis relative to C2-5 (**Figure 5C**).

**Table 1:** Baseline characteristics of the patients with B-ALL in the training cohort.

Variable	All cases (n = 136)	C1 (n = 40)	C2 (n = 32)	C3 (n = 13)	C4 (n = 29)	C5 (n = 22)	*P* value
Age, median (range), y	6.1 (1-21)						0.116^a^
1-10	95 (69.9)	30 (75)	27 (84.4)	7 (53.8)	17 (58.6)	14 (63.6)	
≥10	41 (30.1)	10 (24)	5 (15.6)	6 (46.2)	12 (41.4)	8 (36.4)	
Gender							0.155^a^
Female	69 (50.7)	19 (47.5)	17 (53.1)	5 (38.5)	20 (69.0)	8 (36.4)	
Male	67 (49.3)	21 (52.5)	15 (46.9)	8 (61.5)	9 (31.0)	14 (63.6)	
WBC, median (range), ×10^9^/L	15.8 (1.5-612)						0.130^a^
< 50 × 10^9^/L	91 (66.9)	32 (80)	19 (59.4)	6 (46.2)	18 (62.1)	16 (72.7)	
≥ 50 × 10^9^/L	45 (33.1)	8 (20)	13 (40.6)	7 (53.8)	11 (37.9)	6 (27.3)	
BM blast percentage, median (range), %	91.5 (30-100)	93 (49-100)	90 (74- 99)	87 (65-96)	95 (82-99)	80 (30-96)	0.029^b^
Unknown	10	5	1	1	2	1	
CNS status							0.979^c^
CNS-1	109 (80.1)	31 (77.5)	26 (81.3)	10 (76.9)	25 (86.2)	17 (77.3)	
CNS-2	26 (19.1)	8 (20)	6 (18.8)	3 (23.1)	4 (13.8)	5 (22.7)	
CNS-3	1 (0.7)	1 (2.5)	0 (0)	0 (0)	0 (0)	0 (0)	
NCI risk							0.012^a^
Standard	58 (42.6)	24 (60)	16 (50)	2 (15.4)	8 (27.6)	8 (36.4)	
High	78 (57.4)	16 (40)	16 (50)	11 (84.6)	21 (72.4)	14 (63.6)	
Fusion							
No fusion	56 (41.2)	6 (15)	29 (90.6)	5 (38.5)	7 (24.1)	9 (40.9)	<0.001^a^
*ETV6*-*RUNX1*	29 (21.3)	26 (65)	0 (0)	2 (15.4)	0 (0)	1 (4.5)	<0.001^a^
*TCF3*-*PBX1*	7 (5.1)	0 (0)	0 (0)	1 (7.7)	6 (20.7)	0 (0)	<0.001^c^
*DUX4*-*IGH*	7 (5.1)	0 (0)	0 (0)	0 (0)	5 (17.2)	2 (9.1)	0.005^c^
Mutation							
No mutation	67 (49.3)	26 (65)	11 (34.4)	4 (30.8)	17 (58.6)	9 (40.9)	0.036^a^
*KRAS*	22 (16.2)	6 (15)	8 (25)	5 (38.5)	1 (3.4)	2 (9.1)	0.026^c^
*NRAS*	16 (11.8)	2 (5)	8 (25)	1 (7.7)	1 (3.4)	4 (18.2)	0.039^c^
*IKZF1*	8 (5.9%)	2 (5)	2 (6.3)	2 (15.4)	1 (3.4)	1 (4.5)	0.653^c^
*PTPN11*	6 (4.4%)	0 (0)	3 (9.4)	0 (0)	2 (6.9)	1 (4.5)	0.272^c^
*PAX5*	6 (4.4%)	0 (0)	0 (0)	1 (7.7)	4 (13.8)	1 (4.5)	0.021^c^

WBC, white blood cells; BM, bone marrow; CNS, central nervous system.

^a^Chi-square test; ^b^Kruskal-Wallis test; ^c^ Fisher’s exact test.

## 4 Discussion

Although molecular taxonomies based on RNA-seq data has been proposed in recent years, B-ALL is a heterogeneous disease, especially in terms of the TME, leading to a wide variation in prognosis. However, studies on TME are limited and molecular classification based on the immune profiles is lacking. Our study identified five distinct molecular subtypes based on IRGs by unsupervised clustering, each with different biological characteristics, clinical behavior and prognostic implications. This novel classification advances our understanding of TME of B-ALL and may provide novel potential targets for immunotherapy.

We found that C1 was associated with better prognosis, and C2, C3, C4, and C5 were associated with relatively poor prognosis. To gain a deeper understanding of the differences in survival between subtypes, we performed multiple immune analyses to explore the differences in the immune landscape between the different subtypes. The results showed that C5 with relatively poor prognosis had higher immune and stromal scores, as well as higher CD8 T cell infiltration. This phenomenon appeared to be inconsistent with the current understanding that TME infiltrated with CD8 T cells, NK cells, and M1 macrophages suggests a favorable prognosis (21). However, we found that most of the inhibitory checkpoints (e.g. *BTLA*, *CTLA4*, *TIGIT*) were markedly higher expressed in C5. As we know, these ICPs are expressed on T cells, B cells, NK cells, monocytes, and dendritic cells, and enable tumors to evade immune surveillance, thus leading to growth and spread of tumors (6, 22–24). Based on these results, we can reasonably speculate that although C5 has a high immune cell infiltration, these immune cells may be inhibited by highly expressed inhibitory checkpoints, and thus may not be able to exert their anti-tumor effects well. This may explain why C5 has a relatively poor prognosis. Patients in C5 may benefit from some immune-checkpoint blockade (ICB) therapies such as anti-*CTLA-4*/*PD-1* combination therapy. This is supported by previous (NCT02374333, NCT02906371) and ongoing clinical trials (NCT02879695) reporting that the combination of Pembrolizumab or Nivolumab (PD-1 inhibitors) or Ipilimumab (CTLA-4 inhibitor) with CAR T cells may circumvent CAR-T cell exhaustion within TME, prolong their vivo persistence and improve clinical outcomes (25–27).

Among C1-4, we found that immune and stromal scores of C2 were considerably higher than these of C1, C3, and C4, and that C2 showed remarkably activation of APP and significantly higher scores of activated M1 macrophages. These findings suggested that patients in C2 may have a favorable prognosis, but similar to C5, C2 also had a relatively poor prognosis. Previous studies found that antitumor M1-like phenotype macrophages in the TME may evolve into the pro-tumor M2-like phenotype macrophages under specific circumstances and in the presence of certain stimuli (28, 29). Thus, the higher activated M1 macrophage score at baseline may not adequately indicate favorable patient prognosis. In addition, the relatively poor prognosis of C2 may be caused by the higher number of patients with *NRAS* mutations. A number of previous clinical trials have pointed out a correlation between the genetic alternations with responsiveness to the treatment (30, 31). Irving et al. (32) revealed that in B-ALL, patients with RAS pathway mutations (*NRAS*, *KRAS*, and *FLT3*) are more chemoresistant, and *NRAS* or *KRAS* mutations are common genetic abnormalities in relapsed ALL and are associated with poor prognosis. At the same time, that study reported that mutations in the RAS pathway confer sensitivity to mitogen-activated protein kinase (MEK) inhibitors (e.g. selumetinib), indicating that patients in C2 may benefit from MEK inhibitors.

Furthermore, there was no difference in immune scores between C1, C3 and C4. Notably, we found that compared to the C1, C2, C4, and C5, C3 displayed the significant downregulation of APP. And, albeit insignificantly, a noticeable lower score of class I major histocompatibility complex (MHC-I) was observed in C3. APP is the cornerstones of adaptive immunity, which allows for a set of epitope peptides sampled from the intracellular proteome to be assembled and displayed on MHC-I (33). Following peptide-loading, the MHC-I/peptide complex is transported to the cell surface for presentation to cytotoxic T-cells and NK cells to enable immune surveillance (34, 35). The significant downregulation of APP and the obvious decrease of MHC-I score may lead to immune escape of tumor cells in C3, resulting in patients in C3 having a relatively poor prognosis. Based on the improved understanding of APP, previous studies have proposed innovative therapeutic options to modulate APP, such as the development of proteolysis-targeting chimeras or TCR-like antibodies (35, 36). Additionally, the histone deacetylase (HDAC) inhibitors have been found to activate the expression of MHC-I pathway and enhance antitumor immunity (37, 38). In terms of genetic abnormalities, *KRAS* mutations were strongly associated with C3, suggesting that patients in C3 may also be sensitive to MEK inhibitors. Thus, patients in C3 may benefit from innovative immunotherapies (e.g. TCR-like antibodies), HDAC inhibitors, or MEK inhibitors, or rational combination strategies. In addition, it is worth noting that C4 displayed significant downregulation of cytokine-cytokine receptor interaction. Cytokines and their receptors play critical roles in immunomodulation, inflammatory response, tumor metastasis and other physiological processes (39, 40). Based on the tremendous importance of cytokine-cytokine receptor interactions in TME, cytokine agonists may offer potential and novel therapies for patients in C4 (41).

Among all subtypes, C1 has the best prognosis, which may be attributed to the significantly lowest score of immune suppressive cytokines. After that, we sought to parse out clinical features and genetic abnormalities of patients with different prognosis. Compared with C2-5, C1 displayed a strong tendency toward low WBC level, standard risk and *ETV6-RUNX1* fusion, all of which were indicative of a favorable prognosis in B-ALL.

There are some limitations in this study. First, due to the lack of long-term follow-up data in the cohort, the early treatment MRD status was used as a prognostic surrogate indicator. The long-term prognostic outcomes of patients in these immune subgroups warrant further investigation. Second, due to the lack of sufficient post-treatment samples, we are unable to explore changes in immune cell composition and clonal composition following treatment. This is critical to elucidate how chemotherapy alters the B-ALLs in the different clusters, and it stands as a direction for our future research.

**Figure 6:**
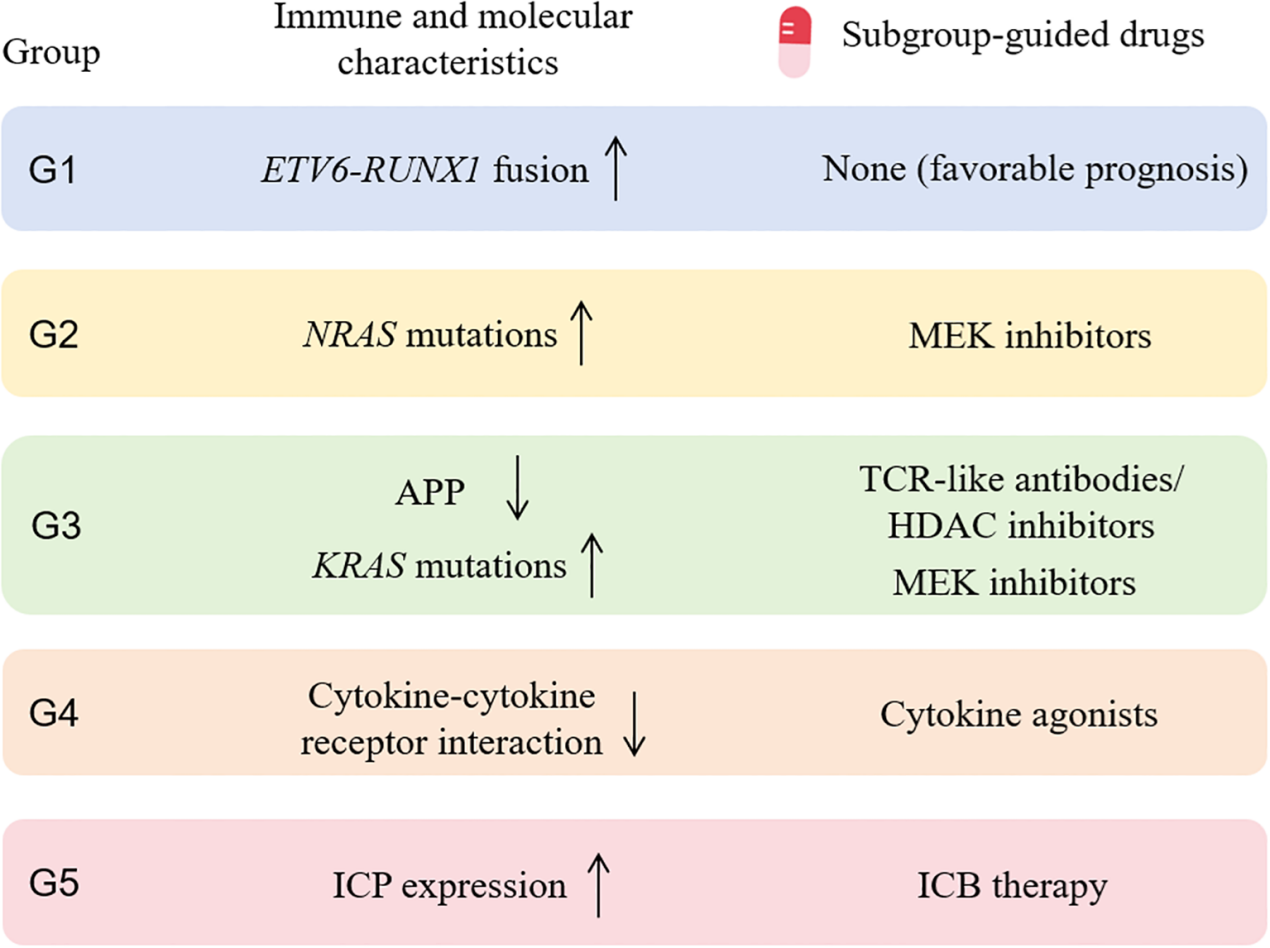
Subgroup-guided agents with potential benefit based on immune and molecular profiling of each subgroup. MEK, mitogen-activated protein kinase; APP, antigen processing and presentation; HDAC, histone deacetylase; ICP, immune checkpoint gene; ICB, immune-checkpoint blockade.

In summary, we identified five immune subtypes of pediatric B-ALL tumors displaying distinct features of immune characteristics, genetic abnormalities and clinical information, which will improve our understanding of heterogeneity of the TME in B-ALL. Importantly, based on the distinct immune and molecular characteristics of each subgroup, we found that G3 and G5 patients may benefit from ICB therapy and TCR-like antibodies, respectively, which directly enhance antitumor immunity through T-cell activation. G2, G3, and G4 patients may benefit from MEK inhibitors, MEK inhibitors/HDAC inhibitors, and cytokine agonists, respectively. While these agents are not classified as immunotherapies, they may collectively remodel the tumor immune microenvironment (TIME), potentially enhancing antitumor immune responses in these patients (**Figure 6**).

## Data Availability

The datasets presented in this study can be found in online repositories. The names of the repository/repositories and accession number(s) can be found in the article/**Supplementary Material**.
